# Co‐Designing the Early Pain Intervention After Knee Replacement (EPIK) Model of Care for People With Persistent Pain After Knee Replacement

**DOI:** 10.1111/hex.70655

**Published:** 2026-03-31

**Authors:** Navneet Chadha, Joshua R. Zadro, Sam Adie, Maria Tchan, Ian A. Harris, Ilana N. Ackerman, Deanne E. Jenkin, Blake F. Dear, Christopher G. Maher, Rachelle Buchbinder, Laurent Billot, Ian D. Cameron, Tracey Gregson, Brenda Luck, Giovanni E. Ferreira, EPIK Study Group

**Affiliations:** ^1^ Institute for Musculoskeletal Health, Sydney Local Health District Sydney New South Wales Australia; ^2^ Sydney School of Public Health, Faculty of Medicine and Health The University of Sydney Sydney New South Wales Australia; ^3^ School of Health Sciences, Faculty of Medicine and Health The University of Sydney Sydney New South Wales Australia; ^4^ Faculty of Medicine & Health, School of Clinical Medicine The University of New South Wales Sydney New South Wales Australia; ^5^ St. George and Sutherland Centre for Clinical Orthopaedic Research Kogarah New South Wales Australia; ^6^ The Centre for Impact & Change Balmain New South Wales Australia; ^7^ Whitlam Orthopaedic Research Centre, Ingham Institute for Applied Medical Research Liverpool New South Wales Australia; ^8^ Musculoskeletal Health Unit and Wiser Health Care Group, School of Public Health and Preventive Medicine Monash University Melbourne Victoria Australia; ^9^ School of Psychological Sciences, Faculty of Medicine, Health and Human Sciences Macquarie University Sydney New South Wales Australia; ^10^ The George Institute for Global Health, Faculty of Medicine, UNSW Sydney New South Wales Australia; ^11^ John Walsh Centre for Rehabilitation Research, Northern Sydney Local Health District Sydney New South Wales Australia; ^12^ The Kolling Institute, Faculty of Medicine and Health The University of Sydney Sydney New South Wales Australia

## Abstract

**Background:**

Approximately one in five Australians who undergo total knee replacement (TKR) experience persistent postoperative pain. There are currently no known effective and cost‐effective multidisciplinary models of care in Australia to address this. We are developing the ‘Early Pain Intervention after Knee Replacement’ (EPIK) model of care, adapted from ‘Support and Treatment After Replacement’ (STAR) care pathway. STAR was developed in the United Kingdom (UK) and shown to be effective and cost‐effective in the UK context. Extensive consumer and clinician engagement informed the development of the EPIK model of care, tailored to the Australian health system and geographical context to address this critical gap.

**Objective:**

To co‐design the ‘Early Pain Intervention after Knee replacement’ (EPIK) model of care for people with persistent pain after TKR.

**Methods:**

We used Experience‐Based Co‐Design (EBCD) methodology to co‐design EPIK, a telehealth model of care providing remote care coordination for people with persistent pain after TKR. Three online Zoom workshops were conducted with key stakeholders, including consumers with lived experience, clinicians (orthopaedic surgeons, physiotherapists, general practitioners, psychologists and a rehabilitation physician), and EPIK investigators. Workshop 1 explored consumer perspectives on the EPIK model of care assessment and follow‐up. Workshop 2 determined the feasibility and roles of clinicians in the EPIK model of care delivery. Workshop 3 brought together consumers, clinicians, and researchers to refine and finalise the EPIK model of care. Using EBCD methodology and inductive framework analysis, themes were derived iteratively. Across consumer and clinician workshops, consensus was reached through facilitated discussions where stakeholders actively contributed to prioritisation activities and iterative refinement of model components.

**Results:**

Twenty‐one individuals participated in the workshops, comprising nine consumers and 12 clinicians from the Australian Capital Territory, New South Wales, Tasmania, and Western Australia, alongside five EPIK Investigators. Workshop 1 with consumers highlighted the need for clearer definition of communication processes, patient education and coaching strategies, and the role of the EPIK care coordinator to ensure consistent information and coordinated support throughout the model of care delivery. Workshop 2 with clinicians focused on telehealth assessments, escalation of clinical pathways, referrals, and psychological support. The final workshop reached consensus on its components and delivery, emphasising the importance of patient advocacy, reassurance and continuity of personalised care.

**Conclusion:**

Through meaningful end‐user involvement, co‐design informed the development of the EPIK model of care. The effectiveness, cost‐effectiveness and safety of EPIK will be evaluated in a randomised controlled trial.

**Patient or Public Contribution:**

Two consumer co‐authors (T.G. and B.L.) guided the development of the preliminary model of care and trial protocol. Consumers with lived experience of persistent pain after TKR participated throughout the co‐design process.

**Trial Registration:**

ACTRN12625001029482p (Australian and New Zealand Clinical Trials Registry). Registered on 17 September 2025.

**Abbreviations**
EBCD: Experience‐Based Co‐Design
EPIK: Early Pain Intervention after Knee Replacement
GP: general practitioner
GRIPP2: Guidance for Reporting Involvement of Patients and the Public 2
NGT: Nominal Group Technique
OECD: Organisation for Economic Co‐operation and Development
OKS: Oxford knee score
STAR: Support and Treatment After Replacement
TKR: total knee replacement
UK: United Kingdom

## 1 Introduction

Osteoarthritis is a chronic joint disease causing pain, disability, and reduced quality of life [1, 2]. Around 2.1 million Australians are affected, with knee osteoarthritis [3] placing a major burden on individuals and the healthcare system. Knee osteoarthritis is a leading cause of functional decline, lost productivity, and the main reason for knee replacement surgery [2, 3, 4]. Australia ranks 5th among the Organisation for Economic Co‐operation and Development (OECD) countries for per capita total knee replacements (TKR), with 69,000 procedures in 2021 [5] projected to exceed 161,000 by 2030, costing $3.4 billion annually [6]. While most patients experience improvements in pain and function [7], around 20% still experience moderate or severe pain 6 months post‐surgery [8]. Similarly to other forms of persistent post‐surgical pain, the aetiology of persistent pain after TKR is multifactorial and poorly understood [9]. Pre‐operative pain intensity and poorly controlled post‐operative pain, surgical complications, and psychosocial factors play a role in the development and perpetuation of persistent pain after TKR [10, 11]. Despite the prevalence of persistent pain post‐TKR, no known effective and cost‐effective multidisciplinary models of care in Australia available to address their needs [12].

A potential model of care to address this is the ‘Early Pain Intervention after Knee replacement’ (EPIK) model. EPIK has been adapted from the United Kingdom (UK) Support and Treatment After Replacement (STAR) pathway, which was shown to be effective, cost‐efficient, and acceptable to both patients with persistent pain after TKR and clinicians in the UK [13, 14, 15]. In STAR, people who had undergone TKR and who had a score of ≤ 14 on the Oxford Knee Score (OKS) pain subscale (indicating pain that negatively impacts their quality of life) were eligible to participate in the STAR care pathway. STAR consisted of an initial in‐clinic assessment, carried out by an extended scope physiotherapist, of potential contributors to the patient's pain, including surgical complications (e.g., stiffness, infection), neuropathic pain, mobility and strength impairments, among others. This extended scope physiotherapist acted as a care coordinator, referring participants to services deemed appropriate to them. Patients also received up to 6 telephone calls over 12 months to monitor progress and reassess the management plan [13, 16]. STAR was designed and tested within the context of the UK's National Health Service and rolled out at eight high‐volume hospitals. The adaptation of STAR into a new context, the Australian health system, required a structured and systematic adaptation process [17]. One of the key adaptations made in EPIK is how the model of care is delivered. While in STAR, the initial assessment, was delivered through an in‐person clinic assessment, EPIK will be entirely implemented via telehealth. This approach was chosen because telehealth is now widespread in Australia, with 22% of Australians using telehealth services in 2024–25 [18]. It also enables the EPIK intervention to reach patients residing in rural and remote areas of Australia.

The adaptation of the UK STAR care pathway into the EPIK model of care occurred in three stages. First, a multidisciplinary team of clinicians and consumers developed an initial draft model. The second stage involved extensive community consultations through 61 interviews (52 clinicians, 9 consumers) [19]. Our qualitative findings underscored a significant gap in care for patients with persistent post‐TKR pain, and provided recommendations on key adaptations to the EPIK model of care to the Australian local context. These insights served as the foundational framework for the third stage of the adaptation process, the co‐design of EPIK with key stakeholders. Co‐design is a collaborative approach involving service users, providers, and stakeholders to ensure meaningful input in shaping decisions [20, 21]. Our study aimed to co‐design EPIK for people with persistent pain after TKR, focusing on the processes for delivery and refinement.

## 2 Methods

**Figure 1:**
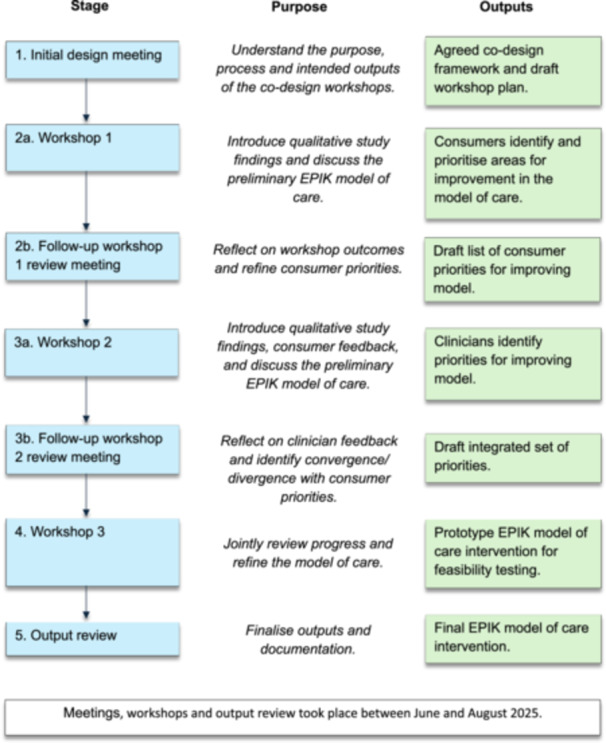
Co‐designing the EPIK model of care: purpose and intended outputs.

We used the Experience‐Based Co‐Design (EBCD) methodology to co‐design EPIK (Figure 1). EBCD is a participatory service improvement methodology grounded in participatory action research principles [22]. It integrates qualitative exploration of lived experience with collaborative service design, bringing consumers and clinicians together to identify key ‘touchpoints’ in care, establish shared priorities, and co‐design solutions that improve both experience and outcomes [23, 24]. Therefore, EBCD was selected for this study because of its emphasis on understanding lived experience, fostering mutual empathy between stakeholders, and enabling shared decision‐making to generate feasible and meaningful improvements in care delivery [25]. The study was approved by The University of Sydney Human Research Ethics Committee (Reference number: 2025/HE000333). All participants provided written informed consent before participating. As there are no established reporting guidelines for co‐design methodology, this study is reported in accordance with the Guidance for Reporting Involvement of Patients and the Public 2 (GRIPP2) short‐form checklist (Supporting Information S1, Section 1) [26].

### 2.1 Study Setting and Sample

We recruited consumers (people with lived experience of persistent pain post TKR) and clinicians (e.g., orthopaedic surgeons, physiotherapists, psychologists, general practitioners, rehabilitation physicians, specialist pain medicine physicians). Consumers were eligible if they currently, or previously experienced persistent pain (≥ 3 months) after TKR. Clinicians were eligible if they were registered to practice in Australia and had managed at least five patients with post‐TKR pain in the past 12 months. Five is an arbitrary number that our team judged to be enough to ensure that clinicians would have minimum exposure to people with the condition of interest.

Consumers and clinicians were recruited using convenience sampling through our existing stakeholder network and passive snowball sampling [27]. Our sampling strategy allowed us to recruit consumers that varied in key aspects such as age, history of persistent post‐TKR pain, geographical location, and education, while clinicians represented different health disciplines. In line with EBCD methodology [22, 25], we aimed for a sample size that would allow for diversity of perspectives while maintaining a group size conducive to active engagement and meaningful co‐design. We capped recruitment at 12 participants per group (consumers and clinicians); this size is consistent with EBCD principles that favour small, manageable group to ensure inclusive and interactive discussions [28, 29, 30]. Participants completed a REDCap questionnaire (Supporting Information S1, Section 2) capturing demographic (e.g., age, gender, ethnicity), clinical (OKS [31]— consumers only), and professional characteristics (e.g., years of experience, clinical practice location—clinicians only).

### 2.2 Co‐Design Process

We adopted a streamlined EBCD process that retained the core methodological stages of EBCD while adapting them to project scope and available resources. Key adaptations included the use of facilitated workshops in place of filmed interviews, and a three‐workshop cycle that moved from separate elicitation of consumer and clinician perspectives to joint prioritisation and design. This approach aligns with recent adaptations of EBCD in diverse settings, including accelerated forms [32] applied to service improvement in chronic disease management [33] and breast and lung cancer services [34]. Workshops were designed and led by an independent facilitator with expertise in co‐design in healthcare (M.T.), who developed and pilot‐tested the co‐design activities (Supporting Information S1, section 3) and set up the online platforms and equipment for the workshops.

### 2.3 Pre‐Design Phase

Findings from our qualitative study demonstrated broad support for EPIK across both consumers and different clinician specialities. These include defining the parameters of the patient–clinician relationship, exploring patient preferences regarding contact by a new clinician (i.e., the EPIK care coordinator) and clarifying the perceived need for engaging the patient's surgeon in the process of care delivered by EPIK. However, the study also identified critical operational and clinical questions that necessitated a collaborative co‐design approach.

An important outcome of our qualitative study was insight into the acceptability of a written management plan structured around short‐ and long‐term goals, to ensure EPIK remains sustainable and patient‐centred. Patients felt that the scope of the EPIK care coordinator should be broadened and encompass education and behavioural support, a substantial modification of the original UK STAR model of care [19].

Regarding clinical diagnostic and triage protocols, decisions were required on the most appropriate screening tools for neuropathic pain (e.g., Douleur Neuropathique 4, PainDETECT) [35, 36] and psychosocial factors (e.g., Pain Catastrophizing Scale, Hospital Anxiety and Depression Scale) [37, 38]. A key discussion point was the triage criteria to guide which patients could be safely managed via telehealth and which would require urgent in‐person review, with clear thresholds for red‐flag symptoms such as suspected infection, wound complications, or acute clinical deterioration.

Additionally, escalation pathways needed to be defined to ensure timely referral to surgeons, general practitioners, physiotherapists, psychologists, or specialist pain medicine physicians when algorithmic pathways were insufficient or patient safety concerns arose. Finally, reporting formats for communicating findings to the participant's general practitioner (GP) and orthopaedic surgeon needed to be standardised, and the patient‐flow diagram refined to integrate EPIK within existing clinical pathways, supporting both safety and continuity of care.

### 2.4 Data Collection

We conducted three workshops via Zoom, each lasting 90–120 min. Workshop activities were tailored to the different stakeholder groups. The purpose of each workshop is summarised in Table 1. For consumers and clinicians, selected elements of the nominal group technique (NGT) were used to structure idea generation, ensure equitable participation, and prioritised options [39]. Following brainstorming, participants independently ranked key ideas; votes were collated and summarised descriptively (frequency counts and rank order) to identify priorities. Decision‐making combined open group discussion with structured consensus techniques. Consensus was defined as broad agreement among participants, achieved through structured, facilitated discussions conducted over multiple rounds. When consensus was reached, results were immediately displayed (e.g., live poll results shared on screen) to provide transparency and allow participants to review outcomes in real time. Items without agreement were documented, further discussed, and revised during subsequent rounds within the same workshop to ensure the final model reflected broad stakeholder input and agreement. These processes operationalised EBCD principles by eliciting lived experience, identifying critical touchpoints, and moving from experience to solution through structured dialogue and co‐creation.

**Table 1:** Purpose of the EPIK co‐design workshops.

Workshop type	Purpose of the workshop
Workshop 1	To explore consumers' needs, preferences, and priorities for assessment, management and follow‐up. Discussions focused on preferred modes of communication and support, and perceptions of what constitutes meaningful recovery.
Workshop 2	To examine the feasibility and clinical applicability of the proposed model of care within existing service structures. Consider professional roles and responsibilities, scopes of practice, interprofessional collaboration and logistical requirements for execution.
Workshop 3	To synthesise findings from the consumer and clinician workshops and facilitate consensus on the structure and components of the EPIK model of care delivery. Consumers, clinicians, and researchers collaboratively refined, prioritised and finalised model elements, ensuring alignment between consumer‐identified needs and clinical feasibility.

Engagement strategies included establishing ground rules for respectful participation and using interactive ranking and consensus‐building activities (Supporting Information S1, Section 3). At each stage, participants were shown how their contributions from earlier workshops had been carried forward into subsequent sessions. Meeting minutes were recorded, audio recordings of all sessions were transcribed verbatim, and screenshots (Supporting Information S1, Section 4) were captured to document artefacts produced during the workshops.

### 2.5 Data Analysis

Workshop discussions were audio‐recorded, transcribed, and analysed using an inductive framework analysis approach [40]. This method was chosen because it accommodates both structured objectives and participant‐led insights, making it particularly appropriate for co‐design research. An initial coding framework was developed by one researcher (M.T.) and refined with input from the EPIK research team (N.C., J.R.Z., S.A. and G.E.F.), based on workshop objectives (e.g., telehealth assessment, care coordination, surgeon involvement). Additional codes were added inductively. Transcripts were coded in Microsoft Word and charted into matrices for comparison across participant groups (consumers, clinicians) and workshop types. Data were analysed separately by group, then mapped into a comparative matrix to identify convergence and divergence, with differences resolved through team discussion. Outputs from consensus activities (polling and prioritisation) and visual artefacts (slides and draft models) were integrated to corroborate findings and document decision‐making. Rigour was enhanced through triangulation, iterative participant feedback, and reflexive discussions between the research team and the external facilitator, ensuring that findings reflected both the structured objectives and participant‐led insights.

## 3 Results

**Figure 2:**
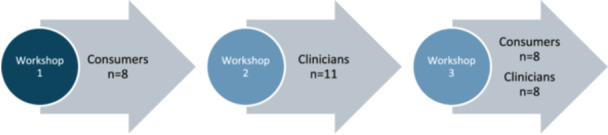
Co‐design participants.

A total of 21 participants took part in at least one co‐design workshop across the study, although not all participants attended both workshops (Figure 2). Our study included nine consumers with lived experience of persistent pain after TKR and 12 clinicians (four orthopaedic surgeons, three physiotherapists, two general practitioners, two psychologists, and one rehabilitation physician). Participants were recruited from the Australian Capital Territory, New South Wales, Tasmania, and Western Australia. Characteristics of included participants are described in Tables 2 and 3.

**Table 2:** Consumer demographics.

Gender	Age (years)	Self‐reported ethnicity	Location of residence	Level of education	Level of income per week	OKS Pain^a^	OKS Function^a^
Female	73	Polish and German	New South Wales	Bachelor Degree or Higher	b	22	18
Female	51	English and Scottish	Western Australia	Advanced Diploma or Diploma	$1600–$2999	19	13
Female	55	Australian and English	Tasmania	Year 12 or equivalent	$1600–$2999	11	10
Female	74	Australian and English	Western Australia	Bachelor Degree or Higher	$800–$1599	18	11
Female	80	Australian	New South Wales	Advanced Diploma or Diploma	$1–$799	17	13
Female	74	Australian	Australian Capital Territory	Bachelor Degree or Higher	$1–$799	17	12
Female	51	English and Scottish	Western Australia	Advanced Diploma or Diploma	$1600–$2999	19	13
Female	68	English and Scottish	New South Wales	Bachelor Degree or Higher	$3000–$5999	19	13
Male	c	c	New South Wales	c	c	c	c

a The OKS is divided into two subscales: pain subscale with a score out of 28 and function subscale with a score out of 20; lower scores on these subscales indicate more severe pain and reduced function, reflecting poorer knee‐related outcomes.

^b^ Indicates that the participant chose not to answer the income level question in the questionnaire.

^c^ Indicates missing data as participant did not complete the questionnaire.

**Table 3:** Clinician demographics.

Variable	(*n* = 12)
Profession, *n* (%)	
Orthopaedic surgeons	4 (33)
Physiotherapists	3 (25)
General practitioners	2 (17)
Psychologists	2 (17)
Rehabilitation physician	1 (8)
Age (years), mean (SD)^a^	47 (9.2)
Gender, *n* (%)	
Male	6 (50)
Female	6 (50)
Years practicing, mean (SD)^a^	
Orthopaedic surgeons	12 (8)
Physiotherapists	21 (8.4)
General practitioners	32 (1)
Psychologists	13 (7.5)
Rehabilitation physician	b
Location of practice, *n* (%)	
Australian capital territory	2 (17)
New South Wales	9 (75)
Tasmania	1 (8)
Occupation setting, *n* (%)	
Public hospital	4 (33)
Private hospital	5 (42)
Other (Aboriginal medical service, University clinic)	3 (25)
Able to provide high quality patient care for patients who have undergone total knee replacement for knee osteoarthritis as a clinician, *n* (%)^a^	
Strongly agree	4 (44)
Agree	5 (56)
Able to provide care aligned with the currently accepted best practice for knee osteoarthritis as a clinician, *n* (%)^a^	
Strongly agree	4 (44)
Agree	4 (44)
Somewhat agree	1 (11)

a Three participants did not complete the questionnaire; summary statistics are calculated from the remaining nine participants.

^b^ Indicates missing data as the participant did not complete the questionnaire.

Analysis identified four overarching themes that captured both shared priorities and points of divergence across all three workshops: (1) communication, education, and coaching, (2) role of the EPIK care coordinator, (3) telehealth assessment and escalation of concerns, and (4) referrals, advocacy, and psychological support (Table 4).

**Table 4:** Key themes and participant quotes from the EPIK co‐design workshops.

Theme	Illustrative participant quotes
Communication, education and coaching	*“That sort of goal setting, and like, really pushing to get that range of motion was key for me.” (Consumer, female)* *“I've experienced as a coaching relationship… involves setting goals…so it's not just having the idea yourself but talking the idea through with another person.” (Consumer, female)* *“The patients…want reassurance and information. They want clear guidance on what pain is normal, what to expect, and consistent education. Patients want coaching and motivation. They'd love to do joint goal setting is really important, reflection and encouragement” (Physiotherapist, female)*
Role of the EPIK care coordinator	*“The coordinator role is an advocate… because very often in these circumstances, you become very demoralised… there was a period for about four months where I never went outside my own front door.” (Consumer, female)* *“The main issue I see with the person from EPIK is… there's no sort of relationship to the patient, so the patient may be a little wary about them recommending they should go and do whatever.” (GP, female)* *“I would just say we could make…the care coordinator…make those recommendations and suggest they discuss them with their surgeon or GP.” (Surgeon, male)* *I would second the open course to be honest, and it's probably a way that you can get everyone getting the same content, so you can be assured that the training is exactly the same, and it's quite accessible.” (GP, female)*
Telehealth assessment and escalation of concerns	*“I've found having agonising detail about how to log on…because there's been so many times where I've just had to be the tech person because a link hasn't worked.” (Physiotherapist, male)* *“If the patient really feels they need to see the surgeon… that is an obvious [reason].” (Surgeon, male)* *“I think that idea of priming is really important. A lot of people have scared scam calls, they just don't answer…I mean, I think the value is that I think having an extra person interested and checking in on them is really important.” (GP, female)*
Referrals, advocacy, and psychological support	*“There won't be a one‐size‐fits‐all… the coordinator should tailor outcomes for people.” (Consumer, female)* *“We have the winner is that…people would love to know personalized referrals who know my circumstances and act as a connector.” (Consumer, female)* *“If there is any perception that they're being referred to a psychological program, and there's any sense that the physical side… is being ignored, I don't think that referral will be received well.” (Psychologist, female)*

### 3.1 Theme 1: Communication, Education and Coaching

Clear, consistent education was a priority across all workshops. Consumers described receiving inadequate information about recovery and wanted reassurance about what pain was normal:

“I lacked the information, the information specifically about what to expect at certain points in your journey.”

(Consumer, female)

Clinicians noted that framing information well was key to consumer confidence and participation. Both groups emphasised that follow‐up should balance structure with flexibility, allowing consumers to choose the mode and frequency of contact:

“The whole idea of having personalised work is to find out from the person what form of communication works best for you.”

(Consumer, female)

Consumers valued coaching‐style support that incorporated joint goal setting and reflection. In contrast, clinicians were more cautious about integrating health coaching into the care coordinator role, noting that delivering effective health coaching would require formal training and certification for EPIK care coordinators, which is not feasible within the time‐limited trial environment. The final workshop confirmed that education, rapport‐building, personalised care and validation should be core functions, supported by templates and scripts for consistency.

### 3.2 Theme 2: Role of the EPIK Care Coordinator

The care coordinator role was consistently valued but interpreted differently by the different groups of participants. Consumers described wanting a connector and an advocate who could actively arrange care and provide motivation:

“It's no good saying to me, you need a physiotherapist. If there's no physiotherapist in 100 kilometres, it's about a knowledgeable person who can act as a connector, and they ring up and advocate on your behalf.”

(Consumer, female)

Clinicians emphasised boundaries, seeing the coordinator as an assessor, communicator, and supporter rather than a substitute for local providers:

“The patient may be wary about someone recommending treatment if they don't know them… it needs to be the surgeon or GP they trust.”

(Surgeon, female)

Consensus was reached on a hybrid model: coordinators would provide structured assessment, educator role, and follow‐up, while GPs and surgeons retained decision‐making authority.

### 3.3 Theme 3: Telehealth Assessment and Escalation of Concerns

Participants agreed that telehealth assessment is feasible but requires clear structure, preparation, and criteria for escalation to in‐person review. Consumers wanted reassurance that their concerns would be taken seriously, and the ability to request escalation when needed:

“If somebody could access the actual surgeon for you and pass on the problem, it would help.”

(Consumer, female)

Clinicians highlighted red flags (infection, wound issues, severe or worsening pain) as automatic triggers for referral back to the surgeon or GP:

“If a patient is struggling, the surgeon needs to know. We expect to see them again to rule out infection, instability, or other surgical issues.”

(Surgeon, male)

The final workshop reached consensus that the model of care should include well‐defined escalation scenarios, balancing patient choice with clinical safety.

### 3.4 Theme 4: Referrals, Advocacy, and Psychological Support

Referrals were a point of divergence. Consumers wanted proactive advocacy and practical help in navigating local services, while clinicians stressed that final referral decisions should remain with the treating GP or surgeon. Structured letters from the EPIK care coordinator back to treating clinicians were proposed as a way of maintaining continuity and trust.

Both patients and clinicians supported the addition of psychological interventions when presented as an adjunct to medical care rather than a substitute:

“It's essential that the medical side is seen to be well managed before referring to an online pain [management]course.”

(Psychologist, male)

Clinicians further supported making pain management courses available to all patients on an optional basis, emphasising that some education is better than none. Consumers highlighted the importance of validating distress and avoiding stigmatising language. Consequently, all participants identified that EPIK care coordinators would play a pivotal role in resource delivery by initiating contact at the 3‐month postoperative mark.

**Figure 3:**
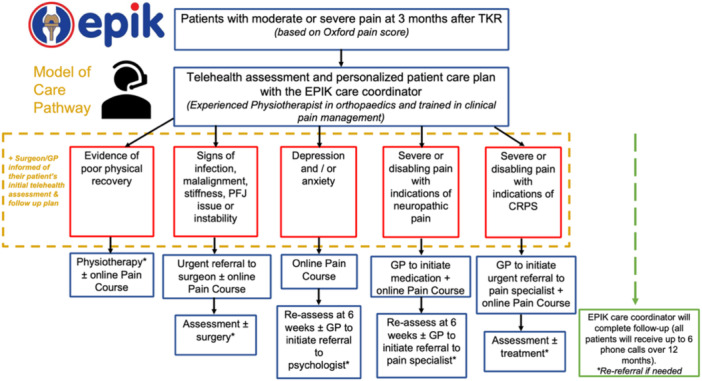
Revised EPIK model of care.

Across the workshops (Table 4), there was strong convergence on the importance of reassurance, education, structured follow‐up, patient‐centred communication, and clear escalation and referral pathways. There was some divergence around the scope of the care coordinator role and the extent of advocacy expected for patients' care needs and preferences. The final workshop reconciled these perspectives into a model balancing consumer agency and support with clinician oversight and safety. The final EPIK model of care (Figure 3) aims to improve patient health outcomes, enhance clinicians' understanding of persistent pain after TKR and its unique intervention needs.

## 4 Discussion

In this study, we adopted a co‐design approach to develop the EPIK model of care intervention pathway, acknowledging its complexity and the importance of collaboratively generating knowledge alongside researchers and key stakeholders [41]. The co‐design process informed critical aspects of the final EPIK model of care, specifically clarifying the EPIK care coordinator role, the way care is coordinated across health disciplines, and how the model can be embedded within existing health services. Ultimately, the EPIK model of care may contribute to transforming approaches to care for people with persistent pain after TKR.

An essential aspect of developing the EPIK model of care was the early identification of key co‐design partners and the cultivation of strong collaborative relationships. This approach ensured that the model was not only evidence‐informed but also acceptable and relevant to end‐users. Engaging consumer stakeholders highlighted priorities that might have been overlooked in a researcher‐ or clinician‐led process, including patient advocacy, tailored referrals, and motivational support. In contrast, professional feedback tended to focus on role definitions and medico‐legal considerations, illustrating how top‐down perspectives alone may fail to capture patient needs [42]. These findings are consistent with previous work in EBCD, which demonstrates that integrating diverse stakeholder perspectives bridges the gap between lived experience and formal expertise [43], ultimately improving the feasibility, acceptability, and uptake of interventions [44]. For example, accelerated EBCD approaches using patient narrative archives have been shown to promote rapid [32], patient‐centred service improvements, while EBCD in chronic disease [33, 45] and cancer care [34] contexts has successfully aligned service redesign with patient priorities and experiences. By embedding consumers as equal partners throughout the development of EPIK, this process exemplifies the application of evidence‐informed EBCD frameworks that integrate research evidence and lived experience to co‐create interventions that are both practical and patient‐centred [44, 46].

All stakeholders identified and agreed on several priority areas for the EPIK model of care delivery. A key focus is the incorporation of personalised care, delivered by clinicians who understand each patient's unique circumstances and can advocate on their behalf. Additionally, clinicians identified a key priority to reframe patient messaging around completion of the online pain management course [47], ensuring it emphasizes coping strategies for managing persistent pain. This will be achieved by equipping EPIK care coordinators with a stronger understanding of pain management, for example through obtaining formal training in pain management.

Our co‐design study reinforces the value of a coordinated model of care in post‐operative orthopaedic practice. Specifically, the findings highlight the importance of clear, structured communication and systematic follow‐up between patients and clinicians. The role of the care coordinator emerged as central in providing education, reassurance, supporting patient motivation, and ensuring safe clinical oversight. These insights align with existing evidence emphasizing informed decision‐making and the benefits of a comprehensive, multidisciplinary approach to patient care [15, 48]. Collectively, these results suggest that embedding care coordination within standard post‐operative pathways warrants further investigation in relation to patient engagement, satisfaction, and recovery‐related outcomes.

### 4.1 Strengths and Limitations

Our study has several strengths. We used a rigorous co‐design methodology based on Experience‐Based Co‐Design (EBCD), emphasizing lived experience, empathy, and shared decision‐making. Involving consumers with persistent pain after TKR, clinicians, and researchers ensured diverse perspectives. Independent facilitation by an EBCD expert promoted neutrality and balanced participation. Iterative feedback loops showed stakeholders how their input shaped later workshops, building trust and validation. The research team (N.C., J.R.Z., S.A. and G.E.F.) and facilitator (M.T.) reflected on how our professional backgrounds could influence the co‐design process. Through regular discussions and oversight of group dynamics, we ensured balanced participation and equal representation of consumer and clinician perspectives that genuinely reflected the shared insights of all stakeholders. Finally, documenting both areas of agreement and points of tension (e.g., the coordinator role) improved the transparency and practical relevance of the model.

Despite broad stakeholder involvement, recruitment may have been influenced by selection bias, with participants likely more motivated about this topic than the wider TKR population. Overrepresentation from New South Wales (*n* = 13) may have limited diversity, and clinicians from pain medicine and rheumatology were not recruited, though both are represented on the EPIK investigators committee. Additionally, consumers who participated in the workshops were mostly female, with 88% from metropolitan areas. All participating consumers had a diploma, advanced diploma, or bachelor's degree, and 50% were from middle‐income households. These demographics show an underrepresentation of men, who represent 40% of patients undergoing TKR in Australia [5], as well as adults with lower educational attainment (e.g., 42% of adult Australians have not completed Year 12) and lower socioeconomic status [49]. While we applied key elements of EBCD to streamline model development, we did not include filmed patient narratives or extended working group cycles, which may have limited the depth of individual storytelling. Conducting workshops virtually via Zoom may have reduced the richness of interaction and excluded participants with limited digital access.

## 5 Conclusion

The co‐design process effectively achieved stakeholder consensus and finalised the EPIK model of care. Key themes shaping the model included communication, education, and coaching to empower patients; the role of the EPIK care coordinator in guiding and coordinating care; telehealth assessment with timely escalation of concerns; and referrals, advocacy, and psychological support to address complex needs. Incorporating these themes and engaging end users ensured the model is patient‐centred, evidence‐informed, and responsive to the needs of people with persistent pain after TKR. The model will next be evaluated in a large randomised controlled trial (ACTRN12625001029482p) that will assess effectiveness, cost‐effectiveness, and safety.

## Author Contributions

**Navneet Chadha:** validation, investigation, data curation, formal analysis, project administration, writing – original draft, writing – review and editing. **Joshua R. Zadro:** conceptualisation, methodology, formal analysis, supervision, writing – review and editing. **Sam Adie:** conceptualisation, methodology, funding acquisition, formal analysis, supervision, writing – review and editing. **Maria Tchan:** methodology, data curation, formal analysis, writing – review and editing. **Ian A. Harris:** conceptualisation, methodology, writing – review and editing. **Ilana N. Ackerman:** conceptualisation, methodology, writing – review and editing. **Deanne E. Jenkin:** conceptualisation, methodology, writing – review and editing. **Blake F. Dear:** conceptualisation, methodology, writing – review and editing. **Christopher G. Maher:** conceptualisation, methodology, writing – review and editing. **Rachelle Buchbinder:** conceptualisation, methodology, writing – review and editing. **Laurent Billot:** conceptualisation, methodology, writing – review and editing. **Ian D. Cameron:** conceptualisation, methodology, writing – review and editing. **Tracey Gregson:** conceptualisation, methodology. **Brenda Luck:** conceptualisation, methodology. **Giovanni E. Ferreira:** conceptualisation, methodology, formal analysis, resources, supervision, writing – review and editing. **EPIK Study Group:** conceptualisation, methodology, writing – review and editing.

## Ethics Statement

All recruitment and data collection procedures were approved by the University of Sydney Human Research Ethics Committee (Reference number: 2025/HE000333). Consent to participate was obtained from all participants.

## Conflicts of Interest

The University of Sydney paid Maria Tchan, a co‐author, for consultancy services related to the design and facilitation of co‐design workshops contributing to the development of the EPIK model of care.

## Supporting information

Supporting_Information.

## Data Availability

The data for this study will not be shared, as we do not have permission from the participants or ethics approval to do so.
