# Supplementary material for: Co‐Designing the Early Pain Intervention After Knee Replacement (EPIK) Model of Care for People With Persistent Pain After Knee Replacement
Source: Health Expect. 2026 Mar 31;29(2):e70655. doi: 10.1111/hex.70655 (PMC13125727; doi:10.1111/hex.70655)
Supplement: Supplementary file 1 — Supporting_Information. [file HEX-29-e70655-s001.pdf]

Supporting Information, Section 1:

Guidance for Reporting Involvement of Patients and the Public 2 - GRIPP 2 short form reporting checklist

**Section and topic**	**Item**	**Reported on page No**
1: Aim	Report the aim of PPI in the study	4
2: Methods	Provide a clear description of the methods used for PPI in the study	4-7
3: Study results	Outcomes – Report the results of PPI in the study, including both positive and negative outcomes	8-10
4: Discussion and conclusions	Outcomes – Comment on the extent to which PPI influenced the study overall. Describe positive and negative effects	10-13
5: Reflections / critical perspective	Comment critically on the study, reflecting on the things that went well and those that did not, so others can learn from this experience	12

PPI=patient and public involvement

Staniszewska S, Brett J, Simera I, et al. GRIPP2 reporting checklists: tools to improve reporting of patient and public involvement in research. BMJ. 2017;358:j3453.

Patient Perspective

Thank you for your interest in our study. We are a team of researchers from several universities across Australia and led by University of Sydney and University of New South Wales researchers who are developing a model of care for people with chronic pain after knee replacement - the EPIK model of care. We are interested in understanding your views on the EPIK model of care, which will inform its design.

What is your age (in years)?

____

What was your sex assigned at birth?

◯ Male
◯ Female
◯ Another term

Please specify another term:

____

What is your postcode?

____

What is your ancestry? (Please provide up to two ancestries only)

☐ English
☐ Irish
☐ Scottish
☐ Chinese
☐ Italian
☐ German
☐ Aboriginal or Torres Strait Islander
☐ Australian
☐ Other

Please specify other:

____

What is the highest level of school or tertiary education that you have completed?

◯ Bachelor Degree or Higher
◯ Advanced Diploma or Diploma
◯ Certificate III or IV (including Trade Certificate)
◯ Year 12 or equivalent
◯ Year 11 or below
◯ Did not go to school

Do you have private health insurance?

◯ Yes
◯ No

What is the total income your household usually receives (pre-tax)?

◯ $6,000 or more per week ($312,000 or more per year)
◯ $3,000 - $5,999 per week ($156,000 - $311,999 per year)
◯ $1,600 - $2,999 per week ($83,200 - $155,999 per year)
◯ $800 - $1,599 per week ($41,600 - $83,199 per year)
◯ $1 - $799 per week ($1 - $41,599 per year)
◯ Nil or negative income

Knee Surgery

Have you had any surgery on your knee because of your knee osteoarthritis?

◯ Yes
◯ No

Which surgery did you have?

☐ Knee arthroscopy (keyhole surgery)
☐ Knee replacement
☐ Other surgery

When did you have your surgery?

____

Please specify other:

____

On a scale of 0 to 10, where 0 represents no pain at all and 10 represents the worst possible pain, please select the number that best describes your pain over the last 4 weeks?

◯ 0 No pain at all
◯ 1
◯ 2
◯ 3
◯ 4
◯ 5
◯ 6
◯ 7
◯ 8
◯ 9
◯ 10 Worst possible pain

Oxford Knee Score

Please answer the following questions based on the past 4 weeks.

1) How would you describe the pain you usually have in your knee?

◯ None
◯ Very mild
◯ Mild
◯ Moderate
◯ Severe

2) Have you had any trouble washing and drying yourself (all over) because of your knee?

◯ No trouble at all
◯ Very little trouble
◯ Moderate trouble
◯ Extreme difficulty
◯ Impossible to do

3) Have you had any trouble getting in and out of the car or using public transport because of your knee? (With or without a stick)

◯ No trouble at all
◯ Very little trouble
◯ Moderate trouble
◯ Extreme difficulty
◯ Impossible to do

4) For how long are you able to walk before the pain in your knee becomes severe? (With or without a stick)

◯ No pain >60 minutes
◯ 16-60 minutes
◯ 5-15 minutes
◯ Around the house only
◯ Not at all - severe on walking

5) After a meal (sat at a table), how painful has it been for you to stand up from a chair because of your knee?

◯ Not painful at all
◯ Slightly painful
◯ Moderately painful
◯ Very painful
◯ Unbearable

6) Have you been limping when walking, because of your knee?

◯ Rarely/never
◯ Sometimes or just at first
◯ Often, not just at first
◯ Most of the time
◯ All of the time

7) Could you kneel down and get up again afterwards?

◯ Yes, easily
◯ With little difficulty
◯ With moderate difficulty
◯ With extreme difficulty
◯ No, impossible

8) Are you troubled by pain in your knee at night in bed?

◯ Not at all
◯ Only one or two nights
◯ Some nights
◯ Most nights
◯ Every night

9) How much has pain from your knee interfered with your usual work? (including housework)

◯ Not at all
◯ A little bit
◯ Moderately
◯ Greatly
◯ Totally

10) Have you felt that your knee might suddenly 'give away' or let you down?

◯ Rarely/never
◯ Sometimes or just at first
◯ Often, not just at first
◯ Most of the time
◯ All of the time

11) Could you do household shopping on your own?

◯ Yes, easily
◯ With little difficulty
◯ With moderate difficulty
◯ With extreme difficulty
◯ No, impossible

12) Could you walk down a flight of stairs?

◯ Yes, easily
◯ With little difficulty
◯ With moderate difficulty
◯ With extreme difficulty
◯ No, impossible

13) Total OKS

____

Clinician Perspective

Thank you for your interest in our study. We are a team of researchers from several universities across Australia and led by University of Sydney and University of New South Wales researchers who are developing a model of care for people with chronic pain after knee replacement - the EPIK model of care. We are interested in understanding your views on the EPIK model of care, which will inform its design.

What is your age (in years)?

____

What is your sex assigned at birth?

◯ Male
◯ Female
◯ Another term

Please specify another term:

____

What is your ancestry? (Please provide up to two ancestries only)

☐ English
☐ Irish
☐ Scottish
☐ Chinese
☐ Italian
☐ German
☐ Aboriginal or Torres Strait Islander
☐ Australian
☐ Other

Please specify other:

____

What is your clinical background?

◯ Medicine
◯ Physiotherapy
◯ Psychology
◯ Exercise physiology
◯ Podiatry
◯ Pharmacy
◯ Chiropractic
◯ Osteopathy
◯ Nursing

What is your area of speciality?

◯ General practice
◯ Rheumatology
◯ Orthopaedic surgery
◯ Anaesthesiology
◯ Occupational and environmental medicine
◯ Emergency medicine
◯ Rehabilitation medicine
◯ Other

Please specify other:

____

Which State or Territory do you spend the most time practicing in?

◯ New South Wales
◯ Victoria
◯ Queensland
◯ South Australia
◯ Western Australia
◯ Tasmania
◯ Northern Territory
◯ Australian Capital Territory

Which clinical setting have you spent the most time practicing in?

◯ Private practice
◯ Public hospital
◯ Private hospital
◯ Other

Please specify other:

____

How long have you been practising for (in years)?

____

On average, how many patients with knee osteoarthritis or with a chronic musculoskeletal condition do you manage/review per week?

◯ 1-4
◯ 5-10
◯ 11-20
◯ 21-50
◯ >50

I am confident that I am able to provide high quality patient care for people who have undergone total knee replacement for knee osteoarthritis:

- ◯ Strongly Agree
- ◯ Agree
- ◯ Somewhat agree
- ◯ Neither agree nor disagree
- ◯ Somewhat disagree
- ◯ Disagree
- ◯ Strongly disagree

I am able to provide care aligned with the currently accepted best practice for knee osteoarthritis

- ◯ Strongly Agree
- ◯ Agree
- ◯ Somewhat agree
- ◯ Neither agree nor disagree
- ◯ Somewhat disagree
- ◯ Disagree
- ◯ Strongly disagree

Supporting Information, Section 3:

EPIK model of care co-design workshop 1

TIME	ITEM
10:00am (15 mins)	1. Welcome & Introductions<br>· Acknowledgement of Country<br>· Acknowledge patient experience, needs and preferences as central to shaping the model of care<br>2. Establish 'ways of working together'<br>3. Explore mindsets for co-design and purpose of today's session
10:15am (15 mins)	**Presentation of the DRAFT EPIK Model of Care**<br>· Focus on when and how support is offered, who is involved, and available supports<br>· Facilitated discussion led by patient questions
10:30am (15 mins)	**Findings from the EPIK Qualitative Study**<br>· Set the scene and describe what we know, and what we are building on today
10:45am (60 mins)	**What does good support look like to you?**<br>Facilitated discussion, using the Nonimal Group Technique<br>1. Invite new ideas before sorting into themes. Prompt questions will be provided using the questions the EPIK team has provided to the facilitator.<br>2. Round robin style discussion and collection of ideas **(Use of Miro, Zoom chat)**<br>3. Group similar ideas together (use EPIK Qual Study themes)*<br>4. Limit each voting round to a single, clear question (e.g., "vote on importance").<br>5. Highlight top ranked items under each theme: if time, ask "why is this on top?" to surface nuance.
11:45am (15mins)	**Wrap up and next steps**<br>Explain how the above activity will feed into refining the EPIK MoC and next steps (Workshop 2 & 3).

EPIK model of care co-design workshop 2

TIME	ITEM
9:00am (10 mins)	Welcome & Introductions<br>· Acknowledge role of clinician as central to success of EPIK model of care
9:10am (15 mins)	**Presentation of the DRAFT EPIK Model of Care**<br>Focus on clinical feasibility, clarity of roles, risks and enablers
9:25am (40 mins)	*"What information, decision supports, and escalation pathways must be in place for the care coordinator to complete a valid, reliable EPIK telehealth assessment and initiate appropriate management or referral?"*<br><u>**Activity 1:**</u> **Assessing the pain causing factors**<br>1. Evidence of poor physical recovery<br>2. Signs of infection, malalignment, stiffness, PFJ issue and instablity<br>3. Depression and/or anxiety<br>4. Severe or disabling pain with indications of neuropathic pain<br>5. Severe and disabling pain with indications of CRPS
10:05am (15 mins)	<u>**Activity 2:**</u> **Referrals and advice**<br>Q. What is the level of involvement/role of the EPIK care coordinator post assessment in terms of the patient care i.e. refer patients to specific health services within their local area and be advocate for the patient to ensure the patient receives the treatment?
10:20am (15mins)	**Wrap up and next steps**<br>Explain how the above activity will feed into refining the EPIK MoC and next steps (Workshop 3).

EPIK model of care co-design workshop 3

TIME	ITEM
9am	**Welcome & Purpose**<br>· Acknowledgement of Country<br>· Purpose of final workshop<br>· Codesign mindsets
9:10am	**Recap of Workshops 1 and 2**<br>**Presentation of the UPDATED EPIK Model of Care**<br>· Show updated to the MoC<br>· Open up for questions<br>*Does any of the language need to be updated to reflect the patient-centred MoC?*
9:30am	**Activity 1 –Testing the EPIK model**<br>*"It is a given that all surgeon's will be informed of the EPIK initial assessment via letter/report.*<br>*In what scenarios, should the EPIK coordinator escalate concerns back to the care team (to be seen in their rooms)?*<br>*What are the cost implications of this?*
10:00am	**Actvity 2 - Referral pathway**<br>"*We hear that the EPIK coordinator can make recommendations, then go to surgeon/GP for referral. What does this look like in practice?"*<br>*From patient perspective, then from clincian perspective.*
If not discussed in the recap section	**Activity 3 - The pain course – where does it sit?**<br>**Show a visual map of EPIK model elements.**<br>*"Where should the pain course sit? Front and centre for all patients, or as an additional option when certain needs are flagged?"*<br>**Output:** Shared decision on whether the course is universal, selective, or tiered.
10:20am	**Final reflections – wrap up and next steps**<br>What must be carried into the trial, and what still requires testing/refinement.

Supporting Information, Section 4:

. What should the service includ 0

- check in - how are you going? 1
- eduction about what to expect in your recovery journey? 2
- referral - ideas on which health care professionals to see 0
- given a choice - have a email/ text and then have a link to talk to someone - led by patient - not set appt 1
- education resources sent out such as recovery plans given out by orthos post surgery - what to expect etc. 1
- coaching relationship - involves setting goals together, (talking through) - with follow up and reflection 2
- personalised resources - who knows my circumstances 1
- personalised referrals - who knows my circumstances - acts as a connector - ring up and advocate on your behalf 3
- communication giving me the information I need - the practical details - when is follow up etc 2
- weekly in the early days 0
- motivation - 'watch' steps per day - tools to motivate 1
- reassurance - the check in - experience of pain - ease anxiety - validating 1
- show me what you've been doing - re exercises - eg. goal setting ROM 2
- resources that are evidence-based and consistent 0

- local knowledge is important
- cost is important
- remedial massage
- physio
- types of equipment - bands, bike, crutches,
- dietitan
- pain specialist
- don't forget your GP - pain relief and support

Ortho

- need to know they are available if I need them +++
- great with surgeon work - not all surgeons have great people skills
- yes to follow up
- reassurance that what you're going through is normal
- they should know that patient is receiving EPIK care coordination.
- 'keep in the loop'
- someone has to pay if surgeon is involved
-

- weekly would be great - varying length
- phone initially - discuss going forward - how moving forward - ask preference
- in pain - last to log on and see someone
- 'horses for courses' - offer numerous communication channels and flexibility around timing
- video vs phone? 'Video is better' - exercises, knee health,
- menu from which you choose - 'such as a drop box'

Polls/quizzes

‹ Back

POLL 1: Escalation back to GP/Surgeon

Poll ended | 1 question | 18 of 19 (94%) participated

1. In what situations should the EPIK care coordinator escalate concerns back to the GP or surgeon? (Multiple choice)

18/18 (100%) answered

Concerns about wound healing or possible infection	(16/18) 89%
Suspected implant or stability issues with the knee	(16/18) 89%
Severe or worsening pain not improving with rehabilitation	(17/18) 94%
Mild knee ache with difficulty walking long distances and up/down stairs	(1/18) 6%
Little progress in recovery after a reasonable time	(11/18) 61%
High levels of distress (e.g., anxiety, low mood, catastrophising) affecting recovery	(6/18) 33%
All patients should be reviewed by their surgeonn	(3/18) 17%
Other (please add in chat)	(4/18) 22%

Polls/quizzes

‹ Back

POLL 2 – Referral Pathway Role

Poll ended | 1 question | 17 of 19 (89%) participated

1. What should the EPIK care coordinator's role be in supporting referrals and communication with the care team (GPs/surgeons)? (Multiple choice)

17/17 (100%) answered

Provide patients with information/recommendations, and the patient follows up	(8/17) 47%
Send recommendations to the GP/surgeon, who decides on next steps	(5/17) 29%
Discuss options with patients, share a summary with GP/surgeon and GP/surgeon initiates referral to pain specialist and/or psychologist as indicated by initial EPIK assessment	(14/17) 82%
Suggest referrals and follow up to check if they happened, while GP/surgeon remains the decision-maker	(7/17) 41%
Other (please add in chat)	(3/17) 18%
